# Tooth replantation with adipose tissue stem cells and fibrin sealant: microscopic analysis of rat’s teeth

**DOI:** 10.1186/s40064-016-2263-9

**Published:** 2016-05-17

**Authors:** Sezin Demirel, Mehmet Emir Yalvac, Sidika Tapsin, Serap Akyuz, Esin Ak, Sule Cetinel, Aysen Yarat, Fikrettin Sahin

**Affiliations:** 1 Department of Pediatric Dentistry, Faculty of Dentistry, Marmara University, Istanbul, Turkey; 2 Center for Gene Therapy, Research Institute at Nationwide Children’s Hospital, Columbus, OH USA; 3 Department of Genetics and Bioengineering, Yeditepe University, Istanbul, Turkey; 4 Department of Histology and Embryology, Faculty of Medicine, Marmara University, Istanbul, Turkey; 5 Department of Basic Medical Sciences, Biochemistry, Faculty of Dentistry, Marmara University, Istanbul, Turkey

**Keywords:** Stem cells, Tooth avulsion, Tooth replantation, Fibrin tissue adhesive, Periodontal ligament

## Abstract

Treatment for dental avulsion cases is early or late replantation of the traumatized teeth. Prognosis of the replanted tooth depends on the level of periodontal injury. Adipose tissue stem cells (ATSCs) were reported to improve periodontal ligament tissue (PDL) regeneration. Fibrin sealant (FS) contains thrombin and fibrinogen to form an adhesive fibrin clot routinely used in surgical procedures. Here, we aimed to investigate the effects of ATSCs + FS treatment on healing of PDL after tooth replantation in a rat model. After 60 min of extraction, maxillary central incisor teeth were replanted with ATSCs + FS. Two months later, the rats were sacrificed and hemimaxilla blocks were dissected out for histological analysis. The results showed that there was a significant improvement in histological findings of ATSCs + FS treated group compared to only FS treated and non-treated groups corresponding to reduced inflammatory resorption and increased new PDL formation. Furthermore, the ankylosis levels were lowered after ATSCs + FS treatment. Singular use of FS improved PDL healing moderately. Our results indicated that ATSCs + FS treatment improves PDL healing after tooth replantation suggesting a new therapeutic potential in the treatment of dental avulsion cases.

## Background

Dental avulsion implies total displacement of the tooth out of its socket. Avulsion of teeth occurs most often in children from 7 to 9 years of age, when the permanent incisors are erupting (Andreasen 1970). Various statistics have shown that avulsion of teeth following traumatic injuries is infrequent ranging from 0.5 to 3 % of traumatic injuries in the permanent dentition (Andreasen 1970). Histologic examination of replanted human and animal teeth has revealed four different healing modalities in the periodontal ligament (PDL): (1) healing with a normal PDL, (2) healing with surface resorption (healing-related resorption), (3) healing with ankylosis (replacement resorption), (4) healing with inflammatory resorption (Andreasen and Andreasen 1992). A normal PDL formation after tooth replantation is difficult to achieve in clinical situations, because the injury results in a minimum lesion in the internal layer of the PDL. The use of substances such as enamel matrix derivative (Emdogain) and Vitamin C for root surface treatment applied before replantation reduced the root resorption not ensuring PDL regeneration (Barrett et al. 2005; Panzarini et al. 2008). Emdogain and low level laser therapies were found to be insufficient to secure an improved PDL regeneration after replantation of tooth (Barrett et al. 2005; Saito et al. 2011). Therefore, there is a need of new therapeutic strategies to enhance PDL regeneration in avulsion cases.

ATSCs can differentiate into bone, cartilage, adipose and neurogenic tissues (Ogawa et al. 2004; Safford et al. 2002; Zuk et al. 2002). There are a number of published clinical applications of ATSCs for the treatment of graft versus host disease (Fang et al. 2006), diabetes mellitus type 1 (Trivedi et al. 2008), Crohn disease (Garcia-Olmo et al. 2009),  rheumatoid arthritis (Ichim et al. 2010), multiple sclerosis (Riordan et al. 2009), tracheomediastinal fistula (Alvarez et al. 2008), calvarial bone defects (Lendeckel et al. 2004), osteoarthritis (Pak 2011), and spinal cord injury (Ra et al. 2011). There are also various applications of ATSCs in plastic surgery (Gir et al. 2012). ATSCs were reported to enhance PDL formation (Tobita et al. 2008). It is not clear how ATSCs exert their therapeutic effect during new PDL formation but it is speculated that ATSCs secrete trophic cytokines which regulates immune responses and stimulation of endogenous progenitor cells increasing the regenerative events (Proksch et al. 2012). Platelet-rich plasma (PRP) derived from patients’ serum has shown success in tooth replantation (Reichert da Silva Assuncao et al. 2011; Tozum et al. 2006) by increasing periodontal tissue regeneration (Tozum et al. 2006). As an alternative to PRP, FS avoids technical difficulties in obtaining PRP from young adults and lowering the inconsistency in therapeutic effect. FS mimics the final stage of the blood coagulation cascade (Thompson et al. 1988) forming a natural bio-scaffold for stem cells to restore the damaged tissue after injury.

In this proof of principle study, we tested if ATSCs + FS administration would be beneficial in tooth replantation in the setting of new PDL formation, prevention of resorption, ankylosis and inflammation. Our results showed unequivocally that ATSCs + FS administration increases periodontal tissue formation after tooth replantation in the rat model. Given that both ATSCs and FS are being used in clinic today, our approach might be translated into clinical trials.

## Methods

### Isolation of ATSCs

Sprague–Dawley rats (4–6 week-old, female) were received an intramuscular injection of xylazine chlorhydrate at dose of 6 mg/kg to attain muscular relaxation and were anesthetized with ketamine chlorhydrate at a dose of 35 mg/kg followed by euthanasia performing cervical dislocation. Adipose tissue surrounding the epididymis was carefully dissected and transferred to the petri dishes (tissue from one rat/dish). The tissues were homogenized by excising into small pieces in 1 ml sterile PBS using a sharp, sterile surgical scissors. Homogenized adipose tissues were transferred into sterile 50 ml falcon tube and rinsed with PBS twice followed by addition of 20 ml collagenase (Sigma) solution at concentration of 0.5 U/ml. The tubes were incubated in a shaker at 37 °C for 45 min. After incubation, collagenase activity was stopped by adding 20 ml 10 % FBS (Fetal Bovine Serum) containing medium followed by centrifugation at 1200 rpm, for 5 min at room temperature. The fat layer and medium supernatant were discarded and cell pellet was washed with 40 ml PBS and then centrifuged again at 1200 rpm for 5 min at room temperature. The cell pellet was resuspended in 2 ml growth medium containing Dulbecco’s modified essential medium (DMEM) supplemented with 10 % FBS, 2 mM of l-glutamine and 1 % of PSF (penicillin, streptomycin and fungizone) solution. 1 ml of cell suspension was transferred to one T150 flask containing 25 ml growth medium and cultured for 10–14 day until the cells reach 80–90 % confluency. Medium was changed every other day and the cells were sub-cultured using trypsin–EDTA solution (1x). All cell culture reagents were purchased from Invitrogen (Invitrogen, Carlsbad, CA, USA).

### Flow cytometry analysis

Briefly, ATSCs were trypsinized and incubated with primary antibodies [CD29 (cat#BD-556049), CD45 (cat#SC-70686), CD90 (cat#SC-53456, SantaCruz Biotechnology Inc., Santa Cruz, CA, USA] in PBS at 4 °C for 45 min. After washing the excess primary antibodies, the cells were incubated with fluorescein iso-thio-cyanate (FITC) conjugated secondary antibodies (SC-2989, Santa Cruz Biotechnology, CA, USA) for 1 h at 4 °C followed by washing with PBS twice and analyzing with flow cytometry. The flow cytometry analysis was carried out using Becton–Dickinson FACS Calibur flow-cytometry system (Becton–Dickinson, SanJose, CA, USA).

### Animals and surgical procedure

Nineteen male Sprague–Dawley rats weighing 250–350 g were used in this study. The experimental protocol was approved by the Animal Research Ethics Committee of Marmara University, Istanbul, TURKEY (Ethic Protocol Number: 30.2011.mar). The animals were fed with a soft diet (increased fat pellet feed) and water ad libitum after surgery. The animals were divided into three groups involving (1) control (4 rats, 8 teeth), (2) FS group (7 rats 14 teeth) and (3) ATSCs + FS group (8 rats, 16 teeth). The animals received an intramuscular injection of xylazine chlorhydrate (Rompun^®^, Bayer Healthcare, Toronto, Canada) at the dose of 6 mg/kg to attain muscular relaxation and were anesthetized with ketamine chlorhydrate (Ketasol, Richter Pharma AG, Wels, Australia) at the dose of 35 mg/kg. A clean operational field was obtained with a 2 % chlorhexidine solution (Clorhexidina s; FGM Dental Products, Dentscare Ltda., Setubal, Portugal) followed by syndesmotomy, luxation and non-traumatic extraction of maxillary central incisors (2 incisors per rat) using extraction forceps of all animals simulating a case of tooth avulsion. Dental papilla located in the apical region was removed. To minimize pulpal infection as stimulus for external root resorption, the pulp tissue was extirpated through a retrograde route with a slightly curved #15 file (Flexofile, Dentsply-Maillefer, Ballaigues, Switzerland). Root canal instrumentation was completed using # 20 or #25 files. Root canals were irrigated with saline (Polifarma Ltda.; Istanbul, Turkey) and fluid was aspirated. Canals were dried with paper points (DiaDent Europe B.V.; Almere, The Netherlands) and obturated with calcium hydroxide paste (Sultan Healthcare, Hackensack, New Jersey, USA) according to the manufacturer’s instructions. Apical accesses were filled with glass ionomer cement (Kavitan^®^ Plus, Spofa Dental, Jicin, Czech Republic). The extracted teeth were held by their crowns, fixed on a red wax plate and kept dry at room temperature (20–24 °C) for 60 min. After 60 min dry storage, the sockets were gently irrigated with saline and the teeth were replanted without any material (control group), with FS (Tisseel Lyo, Eczacıbaşı-Baxter, Istanbul, Turkey) or with ATSCs + FS. In FS group, 200 µl of FS was placed into the alveolar socket before replantation. In ATSCs + FS group, 100 µl thrombin solution containing ATSCs (1 × 10^6^) was mixed with 100 µl protein concentrate and immediately applied to the alveolar socket just before replantation. The replanted teeth did not receive any immobilization. The animals received a single dose of benzathine G penicillin 20,000 IU (Deposilin, I.E. Ula gay, Istanbul, Turkey), via intramuscular injection.

### Histomorphometric analysis

Two months after replantation, the animals received an intramuscular injection of xylazine chlorhydrate at dose of 6 mg/kg to attain muscular relaxation and were then anesthetized with ketamine chlorhydrate at a dose of 35 mg/kg. After checking the depth of anesthesia, rats were sacrificed via cardiac puncture. The right side of the maxilla was separated from the left through the median line with a #15 surgical blade. The maxilla was further sectioned close to the third molar to separate the hemimaxilla containing the replanted tooth.

Samples from maxilla were fixed in 10 % neutral—buffered formalin for 48 h and decalcified in decalcifier solution (Shandon TBD-1, Rapid Decalcifier, Thermo Electron Corp., Pittsburgh, Pennsylvania, USA). Following routine light microscopic preparation, samples were embedded in paraffin wax. Sections with 5 μm thickness were obtained at each 100 μm with a microtome, adding up to 20 sections for each specimen. They were stained with hematoxylin and eosin (H&E) for histomorphometric analyses and were examined under a photomicroscope (Olympus BX51, Tokyo, Japan) on blind study basis. The sections were analyzed with respect to the occurrence of inflammatory resorption, ankylosis, surface resorption and healing with normal PDL. The areas of PDL with small surface resorption on the cementum were marked as surface resorption and the areas with larger resorption with/or areas resorbing into dentine with the inflammatory cells were marked as inflammatory resorption. Areas devoid of PDL; replaced with new alveolar bone apposition and areas with alveolar bone in contact with the tooth were marked as ankylosis. Areas with an undamaged PDL and the ones without resorption were marked as normal PDL healing. For histomorphometric analysis and measurement of the root area affected by the resorption process, the images of the longitudinal root sections were divided into three-thirds (cervical, middle, and apical) using a compass, a ruler and a fine pen. The middle third was selected for the measurements. Images were captured using a digital video camera (Olympus U-TVO.63XC Color Video Camera, Tokyo, Japan) coupled to a Olympus BX51 microscope. The images were analyzed using Image J Program (Image J 1.46r) for determination of healing, resorption areas and ankylosis perimeter. The obtained data in percentages of sites with ankylosis, inflammatory resorption and new PDL formation for both treated and control groups were entered into Excell Software (Excell Software, Microsoft Corp., Redmond, WA, USA) for data quantification.

### Statistical analysis

Mann–Whitney U Test was used for statistical analysis and a p value <0.05 was considered as statistically significant. All statistical analysis was done using SPSS. (Statistical Package for Social Sciences, Version Release 15.0.0.)

## Results

### Characterization of ATSCs

**Fig. 1:**
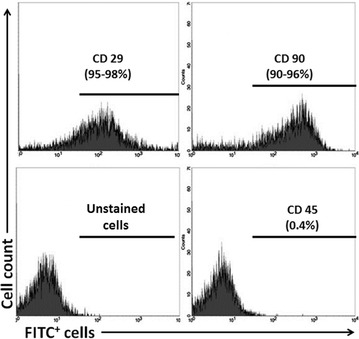
Flow cytometry analysis of ATSCs. ATSCs were analyzed for their surface markers at passage three before transplantation. They were shown to be positive for MSCs markers, CD29 and CD90 but negative for HSC marker CD45 indicating MSCs characteristics of the ATSCs used in this study

When ATSCs reached passage 3, they were checked for the expression of Mesenchymal Stem Cell (MSCs) markers CD29, CD90 and for hematopoietic stem cell marker (HSCs) CD45 using flow cytometry. The results showed that ATSCs isolated from rat adipose stained positive for two main MSCs markers, CD29 and CD90 whereas they were not expressing HSC marker, CD45 (Fig. 1). This indicated that ATSCs used in this study were MSC-like cells when injected into the animals.

### Histomorphometric analysis

**Fig. 2:**
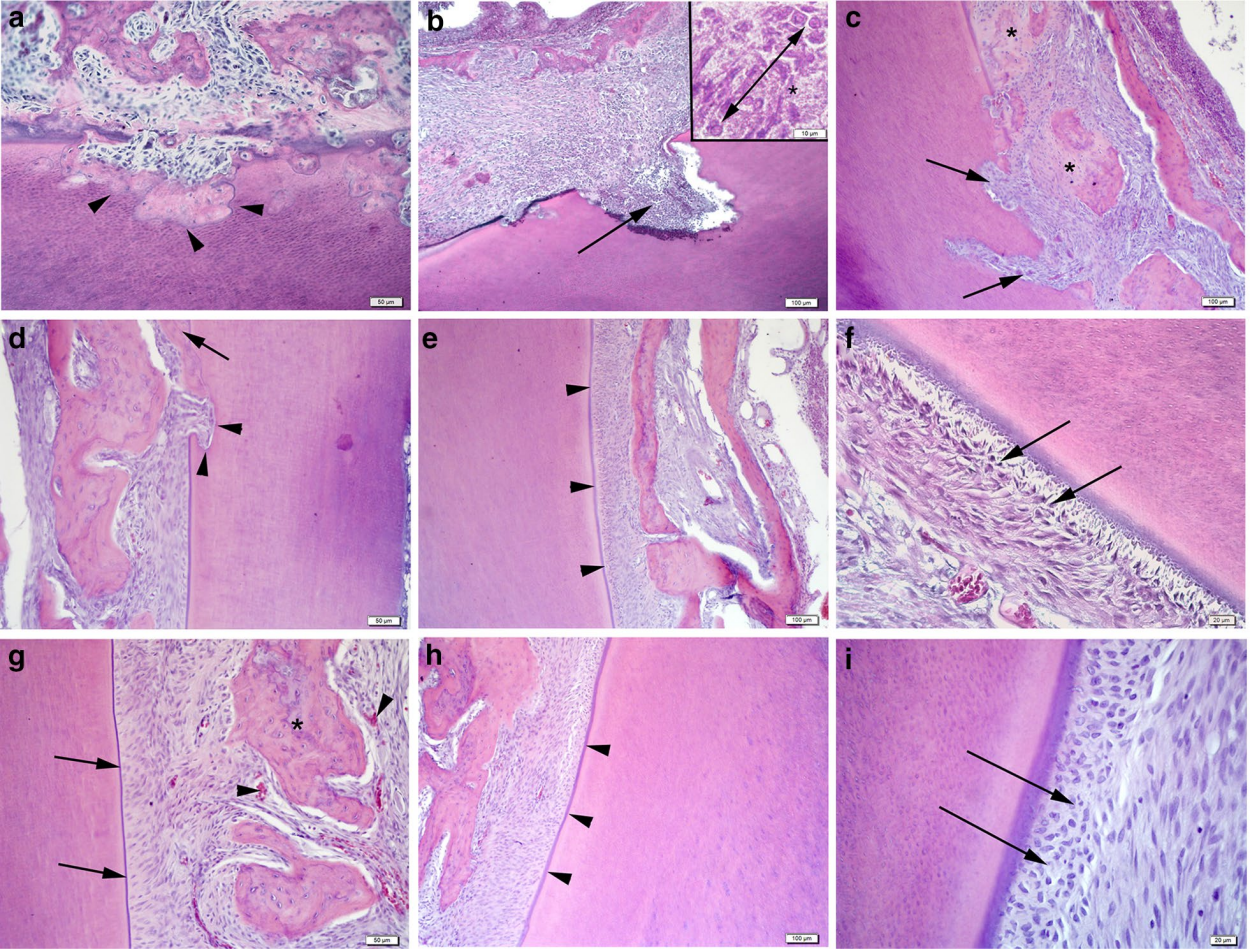
Histomorphometric analysis. Light micrographs of control (**a**–**c**), FS (**d**–**f**) and ATSCs + FS groups (**g**–**i**). **a** In the control group, resorption of cementum and dentin surfaces were filled with newly formed bone in the areas of ankylosis (*arrow heads*). **b** Inflammatory resorption of root surface (*arrow*), lymphocytes (*double headed arrow—insert*) and increase fibroblastic activity (*asterisk—insert*). **c** Inflammatory resorption of surfaces (*arrows*) and areas of ankylosis with new bone formation (*asterisk*). **d** Besides the regular alignment of PDL, the regeneration and resorption on the surfaces (*arrow heads*), some ankylosis (*arrow*) areas near the root surface were prominent. **e** Regenerated regular contour of cement (*arrow heads*). **f** Regular alignment of PDL (*arrows*). **g** Regular alignment of PDL and contour of cement (*arrows*), intense vascularization around the alveolar bone (*asterisk*). **h** Regenerated regular contour of cement (*arrow heads*). **i** Regular alignment of PDL (*arrows*)

Distribution of the percentages of sites with ankylosis, inflammatory resorption and new PDL formation for both treated and control groups were shown in Table 1. Ankylosis in ATSCs + FS group was lowered by 63 and 50 % compared to the control group and FS group respectively (Table 1) (p < 0.05). We observed that FS application showed a trend towards reducing the ankylosis compared to the control group without reaching statistically different levels (p > 0.05) (Table 1; Fig. 2). No inflammatory resorption was detected in ATSCs + FS treated group compared to controls indicating anti-inflammatory roles of ATSCs. Inflammatory resorption was not different (p > 0.05) in FS group and control group in which inflammatory resorption areas were dominantly filled with lymphocytes (Table 1; Fig. 2). The percent of surface resorption was not significantly different among the groups (data not shown).

**Table 1:** Distribution of ankylosis, inflammatory resorption, and new PDL formation

	Control group (teeth, n = 8)	FS group (teeth, n = 14)	ATSC + FS group (teeth, n = 16)
Ankylosis (%)	20.81 ± 17.08	15.57 ± 8.56	7.65 ± 10.24^a, c^
Inflammatory resorption (%)	13.54 ± 13.42	5.01 ± 4.42	0.00 ± 0.00^b, d^
New PDL formation (%)	63.90 ± 16.46	77.99 ± 9.85^a^	92.04 ± 10.23^b, d^

“ %” symbol represents the percentages of sites with ankylosis, inflammatory resorption and new PDL formation for both treated and control groups. Values were given as mean ± standard deviation

*PDL* periodontal ligament, *ATSCs* adipose tissue stem cells

Statistical analysis: *Mann–Whitney U tests*: ^a^ p < 0.05, ^b^ p < 0.001: significantly different from control group; ^c^ p < 0.05, ^d^ p < 0.001: significantly different from fibrin sealant group

In ATSCs + FS treated group new PDL formation improved 31 and 15 % compared to control and FS groups respectively (Table 1). FS improved PDL formation by 19.4 % compared to the control group (Table 1) (Fig. 2e, f) indicating potential therapeutic use of FS in dental replantation. The ATSCs + FS group had a regular contour of cement (Fig. 2g, h). PDL formation and fixation to both cementum and alveolar bone were most prominent in the ATSCs + FS treated group (Fig. 2i).

## Discussion

To date, most replantation studies have focused on factors such as extraoral period, storage media for tooth, timing of endodontic treatment, duration of root canal medication and development of healing complications (Hiremath and Kidiyoor 2011; Lekic et al. 1998; Lin et al. 2000). Andreasen reported that the decisive factor for PDL healing was immediate replantation (Andreasen et al. 1995) indicating that if the teeth are not replanted immediately, they will most probably be lost due to root resorption or infraocclusion (Andreasen et al. 1995). With a limited success Emdogain was used to stimulate new PDL formation by inducing fibroblast migration, proliferation and differentiation into PDL tissue (Wiegand and Attin 2008; Molina and Brentegani 2005; Lam and Sae-Lim 2004). Master regulators of regeneration, stem cells, in combination with biologically safe and biodegradable scaffolds can address the needs for development of new therapeutic techniques in dental avulsion cases. In this regard, we hypothesized that FS guided ATSCs can be utilized in order to increase the success of tooth replantation by improving PDL formation and reducing the inflammatory resorption.

Adipose tissue is an abundant and easily accessible source of adult stem cells. ATSCs can differentiate into different cell types including osteocytes, neurons, myocytes, chondrocytes and adipocytes (Gimble et al. 2007). It has been reported that ATSCs can improve periodontal tissue regeneration (Tobita et al. 2008). The ATSCs were reported to have anti-oxidant capacity by secreting anti-oxidant proteins such as hepatocyte growth factor (HGF), granulocyte-colony stimulating factor (G-CSF), granulocyte–macrophage colony-stimulating factor (GM-CSF), insulin-like growth factor-binding protein (IGFBPs), interleukin-12 (IL-12), platelet-derived growth factor (PDGF), pigment epithelium-derived factor (PEDF), superoxide dismutase (SOD) (Kim et al. 2009). In vitro and in vivo anti-inflammatory effects of ATSCs were associated with their effects on reducing the expression of main inflammatory factors, interleukin-6, tumor necrosis factor-α (Kim et al. 2015) Taken together ATSCs administration in tooth replantation might provide necessary support to reinforce PDL regeneration, healing of the injury site and orchestration of anti-inflammatory effects to prevent inflammatory resorption.

As a blood driven bio-adhesive and bio-scaffold, platelet-rich plasma (PRP) has shown success in tooth replantation (Reichert da Silva Assuncao et al. 2011; Tozum et al. 2006) mainly helping regeneration of bone (Gonshor 2002), tendon (Floryan and Berghoff 2004), and periodontal tissues (Tozum et al. 2006). The success of replantation of teeth is highly dependent on the time of performing replantation after avulsion. The collection of PRP in avulsion cases might delay the necessary replantation procedures. This fact remains as a main obstacle in use of PRP in dental avulsion cases. Furthermore, in the cases of young adults, the patient cooperation might be limited causing inconsistency in therapeutic effect of PRP. Therefore, a ready to use format of PRP such as FS is desirable therapeutic for many surgical operations. FS forms a semi-rigid fibrin clot in the application site based on the activation of fibrinogen to form fibrin by thrombin. FS acts as an adhesive material holding tissues or materials in a required configuration thus providing a 3D scaffold for cells to grow (Radosevich et al. 1997). FS is a clinically proven biomaterial which was shown to promote angiogenesis (Dvorak et al. 1987), local tissue growth and repair (Kram et al. 1988).

In this study we used a rat model. Choosing a rodent model (Lustosa-Pereira et al. 2006; dos Santos et al. 2009; Poi et al. 2007) over a dog, sheep or pig model to test our hypothesis was because of availability and budgetary reasons. To ensure maximum recapitulation of clinical cases, we removed the apical papilla of the teeth which is responsible for continuous growth of incisors teeth in rats as previously described in a number of studies (Saito et al. 2011; Sottovia et al. 2006; Lustosa-Pereira et al. 2006). After treatment, we selected to examine the middle root in all samples considering cervical and apical thirds might be affected during the surgical and endodontic procedures. (Andreasen and Kristerson 1981; Cvek et al. 1990) The avulsed teeth were also left dry for 60 min to simulate a condition of delayed tooth replantation and to make replanted tooth more susceptible to both resorption and ankylosis (Hammarstrom et al. 1989). Histomorphometric analysis showed that control group had remarkably impaired PDL regeneration, well established ankylosis and inflammatory resorption.

ATSCs + FS treatment improved PDL regeneration by 31 % and reduced the ankylosis by 63 % compared to the control group. We did not find any inflammatory resorption in ATSCs + FS group but there were lymphocytes filled inflammatory resorption in FS group. This result indicates anti-inflammatory roles of ATSCs during tooth replantation. Our finding supports the previous reports clearly showing the anti-inflammatory functions of ATSCs in vivo (Gonzalez-Rey et al. 2009, 2010). New PDL formation increased by 19.4 % in FS applied group. This might be attributed to the biological effects of FS such as induction of angiogenesis and improving wound healing (Radosevich et al. 1997) on the remnant PDL fibroblasts on the root surface or in the socket of the tooth through their thrombin receptors (Chan et al. 1998).

In conclusion, in this proof of principle study, we showed that ATSCs + FS treatment significantly improved the success of tooth replantation with normal PDL healing in rats. Although it did not prevent the inflammatory resorption, the use of FS only was shown to improve new PDL formation suggesting that FS itself might have induced endogenous periodontal regeneration to some degree in our model.
